# Prevalence of Eating Disorders and Comorbidity With Depression Among Adolescents in Saudi Arabia: A Cross-Sectional Study

**DOI:** 10.7759/cureus.54366

**Published:** 2024-02-17

**Authors:** Nader Alrahili, Rayan A Alghamdi, Abdulmlk A Alqasem, Afnan Fahad Saleh Alhallafi, Asma A AlFarraj, Shahad K Alghanem, Lina Z Alshalan

**Affiliations:** 1 Psychiatry, Imam Mohammad Ibn Saud Islamic University, Riyadh, SAU; 2 Medicine and Surgery, Imam Mohammad Ibn Saud Islamic University, Riyadh, SAU; 3 College of Medicine, Imam Mohammad Ibn Saud Islamic University, Riyadh, SAU

**Keywords:** eating behaviors, bulimia nervosa, adolescent, depression, eating disorder

## Abstract

Background

Patients diagnosed with eating disorders (EDs) have demonstrated elevated levels of eating psychopathology, including distorted body image, and general psychopathology, encompassing depression, anxiety, impulsivity, and low self-esteem, surpassing anticipated levels. However, the measurement of EDs' prevalence related to depression and mental disorders in Saudi society remains insufficient. There is a clear need for additional studies to establish and substantiate the relationship between these issues and their frequency. In response to this gap, the current study delves into the psychosocial implications of disordered eating in Saudi Arabian adolescents and adults. The primary objective of this study is to contribute to an expanded understanding of the psychosocial effects associated with EDs, shedding light on their prevalence and interconnectedness with mental health concerns among adolescents in Saudi Arabia.

Methodology

A cross-sectional study was conducted in Saudi Arabia among adolescents based on self-report questionnaires, including the Patient Health Questionnaires for Eating Disorders and Diagnostic and Statistical Manual of Mental Disorders, Fourth Edition (DSM-IV) criteria for the presence of EDs. To analyze the obtained data, we calculated the responses from participants who met the following criteria: adolescents (ages 10 to 18 years) from all regions in Saudi Arabia who were ethnically Saudi and included both male and female individuals. Those older than 18 years were excluded from the study.

Results

We collected data from 422 participants; however, 43 responses were excluded because the respondent was under 10 years or over 18 years old. According to the Patient Health Questionnaire 9 (PHQ-9), the prevalence of depression was 83.9%; 36.7% of the participants had severe depression, 23.2% had moderate depression, and 24% had mild depression. According to the Patient Health Questionnaire Eating Disorders Scale, the prevalence of EDs was 23.5%, with the prevalence of binge eating disorder and bulimia nervosa being 14.8% and 8.7%, respectively. We found a significant correlation between EDs and depression, and the severity of depression (P=0.005). The prevalence of depression among patients with EDs was 93.3%: 17.9% of patients with EDs had mild depression, 21.3% had moderate depression, and 54.1% had severe depression.

Conclusion

There is an alarming finding of the high prevalence of both EDs and depression among young adolescents in elementary school, which requires urgent intervention. The study found a significant relationship between EDs and depression: the more severe the depressive symptoms, the higher the prevalence of EDs.

## Introduction

The term "eating disorders" (EDs) refers to a collection of syndromes characterized by eating behaviors and psychological disorders that are also associated with morbid obesity, depressive symptoms, anxiety, abuse of substances, suicidal thoughts, and high rates of mortality and relapse. This significantly affects social interaction and quality of life [1]. EDs include bulimia nervosa, anorexia nervosa, binge EDs, and EDs not otherwise specified [2]. EDs are considered a universal epidemic that is constantly increasing. In recent years, there has been a rise in the occurrence of EDs among adolescents, both male and female, in Eastern countries [3].

Recent research has revealed that EDs are prevalent worldwide [4,5], with EDs occurring more frequently in societies in transition as they acquire Western norms [6,7], highlighting the connection between culture and psychopathology [8]. Throughout the study, the reported prevalence of EDs in the general community ranged from 0.1% to 3.8% [9]. In Western nations, the prevalence of anorexia nervosa is approximately 0.3% among young females, while the prevalence of bulimia is around 1% in the same demographic [10]. Binge ED (BED) prevalence is 1-4%, but 0.3-0.7% of males report EDs [11]. Studies in the Kingdom of Saudi Arabia, Arab countries, and globally show rates of 24% for young female adults, with the most common presentation being anxiety, depression, or a behavioral disorder [5-12]. According to previous reports, subclinical EDs are more common in Eastern nations [13,14]. Populations exhibiting subclinical EDs are at risk of developing additional EDs or potentially progressing to full-blown EDs if effective treatment is not administered appropriately [15]. EDs have a major negative influence on the health of those who suffer from them [16].

Based on behavioral investigations, patients diagnosed with this disorder exhibited higher levels of eating psychopathology (distorted body image) and general psychopathology (depression, anxiety, impulsivity, and low self-esteem) than anticipated [17].

Individuals diagnosed with BED display higher rates of psychopathology, encompassing depression and personality disorders, according to Dobrow; nevertheless, research on the relationship between BED and depression is contradictory [18]. Obese individuals with BED were more likely to be depressed than obese people without BED [19]. According to Borges et al., overweight women with BED, including those with anorexia and bulimia, exhibit heightened concern about their body shape and weight compared to women without BED. Additionally, they reported higher depression ratings and a threefold increased risk of depression compared to overweight women without BED [20].

Although most studies indicate a relationship between depression and EDs, more research is needed to address the limitations reported in these studies. Measuring the prevalence of EDs related to depression and mental disorders in Saudi society is insufficient; more studies are needed to establish the relationship between the two issues and their frequency. The purpose of this research was to expand the scope of knowledge about the psychosocial effects of EDs in the Saudi community. Specifically, we examined whether depressive symptoms and social anxiety increase the risk of EDs among adolescents and adults.

## Materials and methods

Study design and setting

An analytical cross-sectional study was conducted among adolescents in Saudi Arabia to determine the understanding of the psychosocial implications of EDs within the Saudi community. The population included individuals in the age group of 10 to 19 years residing in Saudi Arabia, as acquired from the General Authority for Statistics in the country.

Sample size

The participants involved in this study were in the adolescent age group from a region of Saudi Arabia. The sample size was calculated using online software of Raosoft® with a 95% confidence level and a 5% margin of error, which resulted in a sample size of 385 responses. Data regarding the population of individuals in the age group of 10 to 18 years residing in Saudi Arabia was acquired from the General Authority for Statistics in the country. Every participant had to fulfill the following criteria: male and female individuals from all regions of Saudi Arabia who were ethnically Saudi and aged 10 to 18 years. Individuals who declined to participate in this study were not included.

Sampling technique

Probability sampling was used to choose the participants since it was the most suitable method for selecting a representative sample of our target demographic, which is Saudi Arabian adolescents.

Study population and inclusion and exclusion criteria

This study utilized data obtained from the General Authority for Statistics in Saudi Arabia to investigate the population of individuals within the age group of 10 to 18 years residing in the country. To ensure the accuracy and relevance of the findings, stringent inclusion and exclusion criteria were implemented. Specifically, the study included male and female individuals from all regions of Saudi Arabia who were ethnically Saudi and aged between 10 and 18 years. Participants who declined to participate in the study were not included in the final analysis, thereby maintaining the integrity of the sample population. Individuals known to have depression or mental disorders were excluded. By applying these comprehensive criteria, the study aimed to provide a representative and comprehensive understanding of the targeted age group within Saudi Arabia.

Data collection methods

The target population completed a validated questionnaire that was used to collect data for this investigation. With approval from the responsible authorities, the self-administered computerized questionnaire was sent through social media to the participants. To find out how common EDs are and whether they could be linked to depression, a random distribution of the questionnaire was made. The questionnaire contains three parts. The first part comprises demographic data (age, gender, academic level). The second part comprises the Patient Health Questionnaire-9 (PHQ-9) to screen for depressive symptoms. The third part comprises the Patient Health Questionnaire for Eating Disorders. All scales are translated and validated into the Arabic language.

Data analysis

The data collected were entered and cleaned and the quantitative data were analyzed using the IBM SPSS Statistics for Windows, Version 21 (Released 2012; IBM Corp., Armonk, New York). Qualitative data were presented through frequencies and percentages. Chi t-test, ANOVA, and t-tests were used to assess the relation between different variables. Statistical significance was established at a significance level of p=0.05.

Ethical considerations

The research received ethical approval from the institutional review board of Imam Mohammad Ibn Saud Islamic University, and it adhered to the established standards set by the review board. Prior to their participation, all participants were provided with comprehensive information regarding the study's objectives, and their informed consent was obtained. Confidentiality measures were strictly enforced, ensuring that all responses and personal information of the participants were treated with the utmost privacy and only accessible to the designated study authors.

## Results

In this study, we collected data from 422 participants; however, 43 responses were excluded because respondents were under 10 or over 18 years old. The mean age of the participants was 15.53 (SD=2.3), with 27.2% of participants being 18 years old and 16.6% being 15 years old. Furthermore, 59.6% of the participants were in high school at the time of the questionnaire, and 28.8% were in middle school. Additionally, 73.1% of the participants were female. Moreover, 94.5% of the participants reported living with parents, and 97.1% reported having siblings, with the mean number of siblings being 4.4 (SD=2.4). Furthermore, 12.7% of the participants reported having mental disorders, mainly depression (65.2%) (Table 1).

**Table 1:** Participant Demographics (N=379)

Demographic variable	Value	Count	N %
Age	10	15	4.0%
	11	16	4.2%
	12	16	4.2%
	13	20	5.3%
	14	38	10.0%
	15	63	16.6%
	16	56	14.8%
	17	52	13.7%
	18	103	27.2%
	Mean (SD)	15.53 (2.3)	
Education level	Elementary school	44	11.6%
	Intermediate school	109	28.8%
	High school	226	59.6%
Gender	Male	102	26.9%
	Female	277	73.1%
Living with	Parents	358	94.5%
	Husband/wife	17	4.5%
	Alone	1	0.3%
	Grandparents	3	0.8%
Do you have sibling(s)?	Yes	368	97.1%
	No	11	2.9%
	Mean number of siblings (SD)	4.4 (2.4)	
Have you been diagnosed with a mental disorder?	Yes	48	12.7%
	No	331	87.3%
Type	Depression	30	65.2%
	Generalized anxiety disorder (GAD)	10	21.7%
	bipolar disorder	6	13.0%

**Figure 1:**
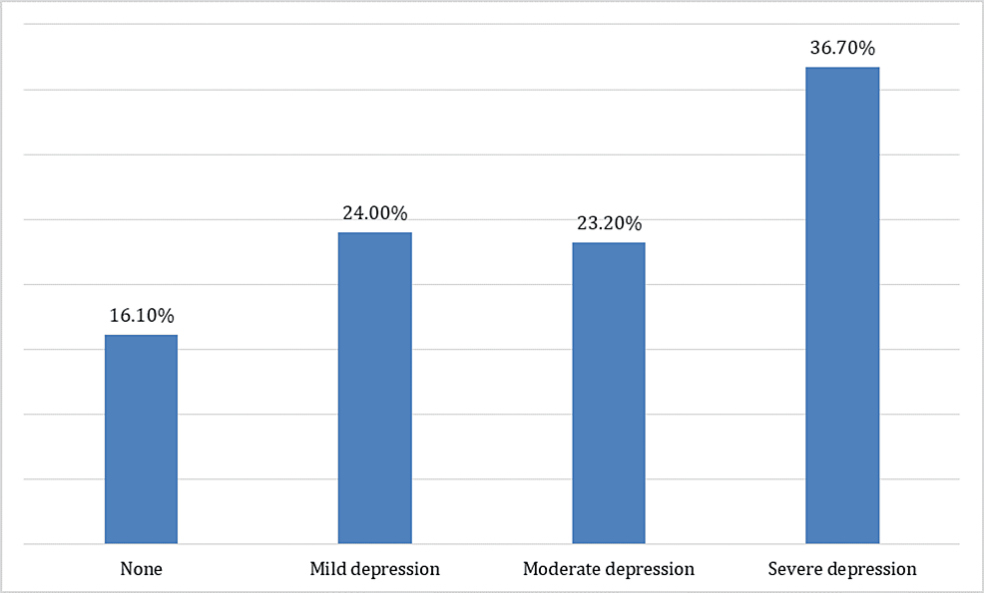
Prevalence and Severity of Depression According to Patient Health Questionnaire 9

According to PHQ-9, the prevalence of depression was 83.9%; 36.7% of the participants had severe depression, 23.2% had moderate depression, and 24% had mild depression (Figure 1).

**Figure 2:**
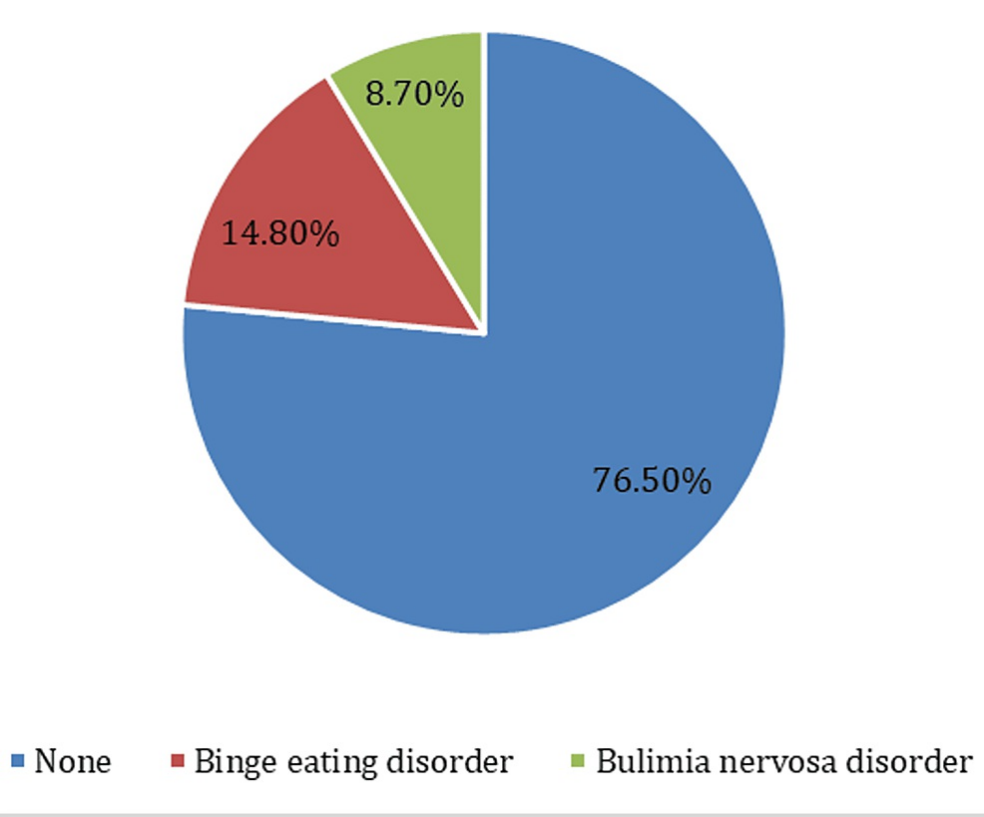
Prevalence of Eating Disorders Among Participants

Depression caused little difficulty on a daily basis concerning work or education for 43.8%, 29.6% had no difficulties because of depression, 15% had serious difficulties, and 11.6% had extreme difficulties. According to the Patient Health Questionnaire Eating Disorders Scale, the prevalence of EDs was 23.5%, with the prevalence of BED and bulimia nervosa being 14.8% and 8.7%, respectively (Figure 2).

Among the participants, we found that 59.9% reported that they often felt that they could not control the quality or quantity of the food they ate, and 31.7% reported that they ate a quantity that most people would find large and unusual. Moreover, 55% of the participants reported fasting for 24 hours in order to lose weight, and 46% exercised for one hour specifically to avoid gaining weight after binge eating. Furthermore, 38.3% of participants reported having no difficulty resulting from EDs during the study, and 34.7% had some difficulty (Table 2).

**Table 2:** Eating Habits and Possible Eating Disorders Among the Participants

Question	Response	Count	N %
Do you often feel that you cannot control the quality or quantity of the food you eat?	No	152	40.1%
	Yes	227	59.9%
Do you eat during any two-hour period an amount of food that most people find large and unusual?	No	259	68.3%
	Yes	120	31.7%
If you answered yes to one of the previous two questions, did this happen again approximately twice a week during the last three months?	No	128	41.7%
	Yes	179	58.3%
In the last three months, have you often done any of the following in order to avoid gaining weight?	Made yourself vomit	34	16.8%
	Took more than twice the recommended dose of laxatives?	20	9.9%
	Fasted – have not eaten anything at all for at least 24 hours?	111	55.0%
	Exercised for more than an hour specifically to avoid gaining weight after binge eating?	93	46.0%
If you checked “YES” to any of these ways of avoiding gaining weight, were any as often, on average, as twice a week?	No	108	47.4%
	Yes	120	52.6%
How difficult is this problem for you when doing your work, studying, performing your responsibilities at home, or getting along with people?	There is no difficulty at all	105	38.3%
	Somewhat difficult	95	34.7%
	Very difficult	49	17.9%
	Unbearably difficult	25	9.1%

The age of the participants had a significant impact on the prevalence of EDs (P=0.032), with the highest prevalence among 10-year-old participants (53.3%) and the lowest among those 17 years old (15.4%). In general, older participants had a lower prevalence of EDs than younger participants. Educational level also had a significant impact on the prevalence of EDs, with the prevalence of EDs being highest among elementary school students (40.9%) and lowest among high school students (19.5%). The prevalence of EDs was slightly higher among males (27.5%) compared to females (22.5%); however, this difference was not significant (P=0.134). We found a significant correlation between EDs and depression, and the severity of depression. More severe depression was associated with a higher prevalence of EDs, especially BED (P=0.005). In general, we found that the prevalence of depression among patients with EDs was 93.3%; 17.9% of patients with EDs had mild depression, 21.3% had moderate depression, and 54.1% had severe depression (Table 3).

**Table 3:** Relation Between Incidence of Eating Disorder and Participants’ Demographic Factors and Depression Severity PHQ-9: Patient Health Questionnaire 9.

		Eating disorder						
		None		Binge eating disorder		Bulimia nervosa disorder		
Demographic variable	Value	Count	N %	Count	N %	Count	N %	P-value
Age	10	7	46.7%	8	53.3%	0	0.0%	0.032*
	11	11	68.8%	2	12.5%	3	18.8%	
	12	9	56.3%	5	31.3%	2	12.5%	
	13	16	80.0%	3	15.0%	1	5.0%	
	14	31	81.6%	4	10.5%	3	7.9%	
	15	48	76.2%	9	14.3%	6	9.5%	
	16	43	76.8%	8	14.3%	5	8.9%	
	17	44	84.6%	4	7.7%	4	7.7%	
	18	81	78.6%	13	12.6%	9	8.7%	
Education level	Elementary school	26	59.1%	14	31.8%	4	9.1%	0.012*
	Intermediate school	82	75.2%	16	14.7%	11	10.1%	
	High school	182	80.5%	26	11.5%	18	8.0%	
Gender	Male	74	72.5%	21	20.6%	7	6.9%	0.134
	Female	216	78.0%	35	12.6%	26	9.4%	
Living with	Parents	277	77.4%	52	14.5%	29	8.1%	0.361
	Husband/wife	10	58.8%	3	17.6%	4	23.5%	
	Alone	1	100.0%	0	0.0%	0	0.0%	
	Grandparents	2	66.7%	1	33.3%	0	0.0%	
Do you have sibling(s)?	Yes	281	76.4%	54	14.7%	33	9.0%	0.572
	No	9	81.8%	2	18.2%	0	0.0%	
PHQ 9 categories	None	55	90.2%	4	6.6%	2	3.3%	0.005*
	Mild depression	75	82.4%	11	12.1%	5	5.5%	
	Moderate depression	69	78.4%	13	14.8%	6	6.8%	
	Severe depression	91	65.5%	28	20.1%	20	14.4%	

## Discussion

EDs predominantly manifest during the late stages of adolescence or early adulthood, but there is limited data regarding the prevalence of EDs among younger adolescents. EDs have been observed to be linked with the physical, social, and psychological development of adolescents [21]. Moreover, the COVID-19 pandemic has been linked to a rise in the prevalence of psychiatric disorders [22]. Psychiatric disorders may prompt individuals to adopt coping mechanisms that involve using eating as a means to shift their attention away from feelings of anxiety and distress. As a result, this can lead to changes in their eating habits and behavior [23].

The core purpose of this study was to assess the prevalence of EDs and investigate their comorbidity with depression among adolescents in Saudi Arabia.

In this study, the prevalence of EDs among adolescents aged between 10 and 18 years was 23.5%, with the prevalence of BED and bulimia nervosa being 14.8% and 8.7%, respectively. This is less than what was reported in prior research among adolescent schoolgirls in Abha City, Saudi Arabia, which reported a prevalence of EDs of 34% [24]. However, our findings exhibited similarity to the outcomes of previous studies conducted in various regions of the kingdom, including 24.6% among schoolgirls in Riyadh [25], 26.1% of adolescent girls in Makkah [26], and 25.47% among female adolescents (aged 15-19) in Arar City [27]. The prevalence of EDs observed in this study falls within a similar range as reported in a previous study, where the prevalence of EDs in Arab countries ranged between 16.2% and 42.7% [28]. A systematic review comprising 22 studies conducted across nine Arab countries reported a prevalence of 26.94% in adolescents of both genders. Notably, the study revealed that the highest prevalence of EDs was observed among adolescents from Saudi Arabia and the UAE [29]. In Western countries, the prevalence of EDs varied across different regions, with reported rates ranging from 0.4% in Spain to as high as 33% in Australia [30,31].

In our study, we identified certain significant factors that were linked to a higher prevalence of EDs, such as being of a younger age and attending elementary school. On the other hand, the study showed that sex was not significantly associated with the prevalence of EDs. In a prior study, researchers reported that older age and a higher educational level were linked to a higher prevalence of EDs, which contrasts with the findings of our study [24-34]. The effect of age has been reported in several previous studies [23-33]. Our results showed that there is a new time bomb, considering the increasing prevalence of EDs among younger adolescents, which may affect their health and development. Therefore, there is an urgent need to develop strategies and programs to increase awareness among parents and students in elementary schools about healthy eating habits and to encourage these populations to exercise and develop good habits other than eating. In our study, we did not observe a significant difference in the prevalence of EDs between sexes; however, the prevalence was slightly higher among male participants. This contrasts with the results of several previous studies that consistently demonstrated a significantly higher prevalence of EDs among females compared to males [24-29,35].

One of the main objectives of this study was to investigate the elevated prevalence of depression among adolescent participants. The prevalence of depression in this group was recorded at 83.9%, with 36.7% experiencing severe depression, 23.2% showing moderate depression, and 24% presenting with mild depression. This is similar to the high prevalence of depression reported in a previous study conducted among 1,245 high school students in the Qassim region, where the prevalence of depression using the PHQ-9 was 74%, with 34% mildly depressed, 24.6% moderately depressed, and 15.4% severely depressed [36]. In a separate study involving 490 high school students in the Taif region, the prevalence of depression among adolescents was reported as 67.3%, with 32.7% found to be mildly depressed, 22.4% moderately depressed, and 11% severely depressed [37]. These findings demonstrate a significantly higher prevalence of depression compared to other studies conducted among adolescents aged 10 to 18 years in different regions. For instance, studies conducted in rural areas of Haryana, India, reported a depression prevalence of 20.6% [38], while a study in Ethiopia found a prevalence of 28% [39]. Additionally, another study conducted in India reported a depression prevalence rate of 3.7% [40]. The high prevalence of depression in our study should be alarming, as there is an urgent need to act to control this prevalence among this important population.

Overall, our findings revealed a notably high prevalence of depression among patients with EDs, with 93.3% of these patients experiencing depression. Among the patients with EDs, 17.9% were mildly depressed, 21.3% had moderate depression, and a significant proportion, 54.1%, presented with severe depression. This is higher than reported by Elmasry and Khali (2018) among 2,000 university students, where 42.4% of students with EDs (471) had depression, 36.5% had mild depression, 57.5% had moderate depression, and 6% had severe depression [41]. Moreover, Giovanni et al. (2011) reported that 19.5% of the patients with EDs suffered from major depression, whereas 48.7% reported clinically significant depressive symptoms [42]. Eisenberg D et al. (2011) reported a lower incidence of depression; the prevalence of depression among students with EDs was 22.8% [43]. Furthermore, we found a significant correlation between EDs, depression, and depression severity. A stronger correlation was observed between more severe depression and a higher prevalence of EDs, particularly BED (P = 0.005). These findings are consistent with numerous prior studies, including those conducted by Sander et al. (2021), which found a significant relationship between depression and EDs, particularly among individuals aged 12-25 [44]. Singleton et al. (2019) also reported a significant association between EDs and lower health-related quality of life, coupled with a higher prevalence of depression [45]. Moreover, Paans et al. (2018) reported that depression is linked to increased emotional and uncontrolled eating as well as reduced cognitively restrained eating. Additionally, somatic depressive symptoms were associated with heightened appetite and weight gain and exhibited a stronger connection with unhealthy eating patterns compared to other symptoms [46]. This association was observed to be significant in both directions, suggesting that the increased prevalence of depression could be a contributing factor to the higher prevalence of EDs, or conversely, the higher prevalence of EDs could be a contributing factor to the increased prevalence of depression observed in this study.

This study had certain limitations that should be acknowledged. One limitation pertains to the use of self-reported questionnaires, which could introduce personal bias and may not fully capture medical conditions in a validated manner; however, clinical assessment is essential. Additionally, the study involved young participants, raising the possibility that some respondents might have faced difficulty comprehending the questions or answered in a random manner. Moreover, the distribution of the questionnaire online could have introduced sampling bias, as older participants who used these applications more frequently might have been overrepresented.

## Conclusions

In summary, EDs encompass a range of syndromes marked by irregular eating patterns and concurrent psychiatric disorders, accompanied by depressive symptoms, exerting a substantial impact on both quality of life and social interactions. Recognizing the pressing need for timely intervention, our study delved into revealing a causative link between EDs and depression. The prevalence of EDs exhibited a notable escalation corresponding to the severity of depressive symptoms. Moreover, our findings underscored the pivotal role of age in influencing the prevalence of these conditions, as younger adolescents emerged as a distinct risk group susceptible to both EDs and depression. Despite recent initiatives to gauge the prevalence of EDs in correlation with depression and mental disorders within Saudi society, current efforts remain insufficient. Further comprehensive studies are imperative to substantiate the intricate relationship between these two issues and delineate their frequency accurately.
